# Diagnostic management of patients reporting symptoms after wheat ingestion

**DOI:** 10.3389/fnut.2022.1007007

**Published:** 2022-10-06

**Authors:** Andrea Costantino, Gloria Maria Aversano, Giovanni Lasagni, Veronica Smania, Luisa Doneda, Maurizio Vecchi, Leda Roncoroni, Elide Anna Pastorello, Luca Elli

**Affiliations:** ^1^Gastroenterology and Endoscopy Unit, Fondazione IRCCS Ca’ Granda Ospedale Maggiore Policlinico, Milan, Italy; ^2^Department of Pathophysiology and Transplantation, University of Milan, Milan, Italy; ^3^Department of Internal Medicine, ASST Grande Ospedale Metropolitano Niguarda, Milan, Italy; ^4^Department of Allergology and Immunology, ASST Grande Ospedale Metropolitano Niguarda, Milan, Italy; ^5^Department of Biomedical, Surgical and Dental Sciences, University of Milan, Milan, Italy; ^6^Allergology, Casa di Cura Ambrosiana, Cesano Boscone, Italy

**Keywords:** celiac disease, wheat allergy, gluten sensitivity, gluten-free-diet, food allergy, gluten, wheat-sensitive IBS

## Abstract

Many patients report symptoms after wheat ingestion experiencing a wide spectrum of clinical manifestations. Three possible diagnoses have been recognized: celiac disease (CD), wheat allergy (WA), and non-celiac (gluten) wheat sensitivity (NCGS/NCWS). CD is a chronic immune-mediated disease of the small bowel caused by exposure to dietary gluten in genetically predisposed individuals, with a prevalence of approximately 1%. It is characterized by mucosal inflammation and atrophy following exposure to gluten and improvement after gluten withdrawal. Food allergies are immunological responses to a food antigen. WA is the expression of an immunologically mediated process that can be immunoglobulin E (IgE) or non-IgE mediated; its many symptoms include urticaria/angioedema, asthma, rhinitis, and anaphylaxis. NCGS/NCWS is characterized by gastrointestinal and/or extra-intestinal symptoms after ingestion of gluten-containing food in subjects not affected by CD or WA. The aim of this review is to help physicians and nutritionists diagnose the cause of symptoms reported after wheat ingestion, thus avoiding patient frustration, inappropriate testing, and incorrect or missed diagnoses. An algorithm for the diagnostic approach in these patients is provided, to help to diagnose CD, WA, NCGS/NCWS or to identify possible functional disorders as the wheat-sensitive irritable bowel syndrome. A personalized approach, regular follow-up, and the help of a skilled healthcare professional are mandatory for patients with symptoms following wheat ingestion is provided. A gluten-free-diet is often recommended for patients with self-reported gluten/wheat-dependent symptoms; for patients with symptoms similar to those of functional diseases while there is evidence that a low-FODMAP diet could be the first option.

## Introduction

Over the last few decades, there has been much interest in sensitivity to or intolerance of wheat and its systemic and gastrointestinal clinical manifestations (1–3). However, confusion about the topic among clinicians and patients may lead to inappropriate testing and missed or incorrect diagnosis. The term food intolerance describes a large array of food-triggered complaints that do not involve the immune system and are experienced by up to one-fifth of the population (4). Lactose intolerance is common among the food intolerance (5, 6). Typical symptoms of food intolerance are mainly gastrointestinal and include gas, distention, bloating, nausea, and changes in stool consistency.

The global incidence of food allergies is rising, especially in industrialized countries. This increase is probably multifactorial, with changes in lifestyle and behavior playing a significant role. The hygiene hypothesis suggests that our environment has become “too clean” and the immune system may overreact unexpectedly to food proteins and create allergic responses (7). Many scientific studies are currently investigating the possible role of our gut microbiome (8, 9).

Many patients report symptoms after eating certain foods, but it is thought that the mechanism is not related to food intolerance or to allergy. Patient exposure to specific foods may also generate less specific and more diffuse symptoms, often described as food sensitivity. Patients frequently suspect the trigger is a gluten-containing food. The exact physiopathology is often unclear (3). In patients reporting symptoms following ingestion of gluten-containing food, it is essential to confirm or exclude celiac disease (CD), a common immune-mediated disease which affects approximately 1% of the population (10).

Celiac disease is a state of heightened immunological responsiveness to ingested gluten in some genetically susceptible individuals. The diagnosis is very important since lifelong adherence to a strict gluten-free diet (GFD) results in an optimal clinical outcome in the large majority of patients, although some report persistent symptoms despite following a GFD (11). If CD is excluded, the presence of certain symptoms (e.g., swelling, itching or irritation of the mouth/throat, hives or itchy rash of the skin, difficulty breathing) should lead clinicians to investigate a possible wheat allergy (WA). In case of exclusion of both CD and WA, non-celiac gluten (or wheat) sensitivity (NCGS/NCWS) should be considered in patients with reported symptoms only after gluten ingestion (12).

This review aims to present current evidence to help healthcare professionals—such as physicians or nutritionists—follow the correct diagnostic approach and proper algorithm for managing patients presenting with symptoms after wheat ingestion.

## Possible diagnosis in patients reporting symptoms after wheat ingestion

### Celiac disease

Celiac disease is a chronic immune-mediated disease of the small bowel caused by exposure to dietary gluten which is composed by the related proteins glutelin and prolamin in genetically predisposed individuals. It is also known as gluten-sensitive enteropathy. The estimated global prevalence based on serological studies is approximately 1% (higher in Europe and North America) (13–15). It is diagnosed more frequently in females (F:M ratio of 2:1) and has a bimodal age at the diagnosis distribution, with an initial peak in the first 2 years of life and a second peak in the second or third decade (14).

Celiac disease is characterized by mucosal inflammation, villous atrophy, and crypt hyperplasia, which occur after exposure to dietary gluten and improve after withdrawal of gluten from the diet (11). Transglutaminase 2, the celiac autoantigen expressed in the intestinal mucosa, modifies immunogenic gluten peptides through deamidation of some charge-neutral glutamine residues, yielding negatively charged glutamic acid residues. This modification promotes gluten peptide presentation by human leukocyte antigen (HLA) DQ2 or DQ8 molecules on mucosal antigen-presenting cells and enables the activation and expansion of gluten peptide-specific CD4+ type 1 helper T cells and the secretion of proinflammatory cytokines. This process leads to villous atrophy and crypt hyperplasia and to B-cell differentiation and the production of transglutaminase 2 IgA (16).

It is crucial to differentiate CD from NCGS/NCWS because of the risks of nutritional deficiency and complications associated with CD. It is also important to differentiate CD from NCGS/NCWS since only for CD patients the adherence to a strict life-long GFD is necessary, considering the difficulty and the costs maintaining it (12, 17).

#### Diagnosis of celiac disease

While thereal benefit of general population screening to detect asymptomatic CD has not been demonstrated (18), appropriate tests to diagnose or exclude CD in patients reporting typical symptoms is essential.

Diagnosis is based on serological testing in adults with suggestive gastrointestinal symptoms including chronic or recurrent diarrhea, signs of malabsorption, unexpected weight loss, abdominal pain, and distension or bloating, or laboratory evidence suggestive of CD such as iron deficiency anemia, folate or vitamin B12 deficiency, and persistent elevation in serum aminotransferases. CD should also be investigated if patients report extra-intestinal signs or symptoms such as fatigue, recurrent headaches, recurrent fetal loss, low birthweight offspring, persistent aphthous stomatitis, dermatological manifestations, metabolic bone disease, or premature osteoporosis (19).

**FIGURE 1:**
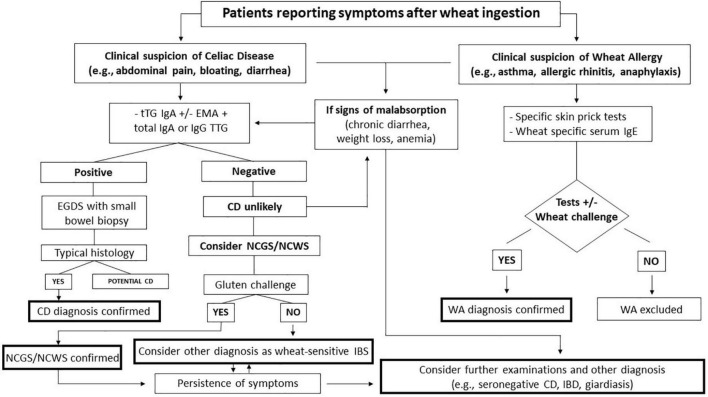
Flow chart of the management of patients reporting symptoms after wheat ingestion. CD, celiac disease; EGDS, esophagogastroduodenoscopy; EMA, endomysial antibody; NCGS/NCWS, non-celiac gluten-wheat sensitivity; tTG; transglutaminase; WA, wheat allergy; IBS, irritable bowel syndrome.

Patients with a very high probability of having CD (e.g., those with diarrhea with features of malabsorption, gastrointestinal signs, and chronic symptoms with a family history of CD) should undergo serological testing and duodenal biopsy to determine whether they have a rare form of seronegative CD or whether their symptoms have another cause (e.g., autoimmune enteropathy, giardiasis, Whipple’s disease). All testing for CD should ideally be performed while the patient is on a gluten-containing diet (19). The diagnostic approach for adults is shown in Figure 1.

Transglutaminase 2 (tTG)-IgA antibody is the single preferred test for the detection of CD in adults due to its high sensitivity and specificity (20–22). In addition, total IgA levels should be measured since CD is associated with IgA deficiency in 2–3% of patients (23). In patients with IgA deficiency, tTG IgG-based testing. should be performed. The tTG-IgA and endomysial (EMA)-IgA antibody tests have similar sensitivity and both tests have high specificity (19). Deamidated gliadin peptide (DGP)-IgG may be performed for children <3 years old with a good diagnostic accuracy in this setting.

Adult patients with positive serological testing should undergo an upper endoscopy with small bowel biopsy to confirm the diagnosis. Multiple biopsies of the duodenum (at least four oriented biopsies of the post-bulbar duodenum) are recommended.

Histological features of CD range from mild alteration characterized only by increased intraepithelial lymphocytes to severe atrophic mucosa with complete loss of villi and crypt hyperplasia (24). The histological severity of intestinal lesions in CD is graded using the Marsh-Oberhuber classification (25). Subtotal or total atrophy (Marsh type 3 lesion), while not pathognomonic for CD, is diagnostic in association with positive serology.

Endoscopic features suggestive of CD have low sensitivity (59–94%), but the reported specificity ranges from 92 to 100%. These findings may be seen with other disorders such as giardiasis, autoimmune enteropathy, and HIV infection (26).

Celiac disease is strongly associated with the HLA class II genes HLA-DQ2 and HLA-DQ8 located on chromosome 6p21. Approximately 95% of individuals with CD express DQ2 heterodimer, while the remainder express HLA-DQ8, with a perfect negative predictive value for CD close to 100% in the absence of these genes. However, they are necessary but not sufficient for the diagnosis. Genetic testing should not be used routinely for the initial diagnosis of CD, but can be used to rule out CD in some clinical situations including Marsh 1–2 histology in seronegative patients, for the evaluation of patients not tested for CD before starting a GFD, and when the results of celiac-specific serology and histology are discrepant (19).

A food challenge should be considered if patients are already on a GFD and carry genetic susceptibility to CD: it is important to carefully evaluate the clinical response and patients should undergo serological tests and duodenal biopsy if necessary (19).

Only for children, diagnosis without an intestinal biopsy is an option for those young patients who have all three following characteristics: (a) tTG-IgA >10 times the upper limit of normal; (b) positive EMA-IgA results; and (c) symptoms compatible with CD, as outlined in the guidelines of the European Society for Paediatric Gastroenterology, Hepatology and Nutrition (ESPGHAN) and the British Society for Paediatric Gastroenterology, Hepatology and Nutrition (BSPGHAN) (27, 28). On contrast, duodenum biopsy is necessary for adults also because a baseline histology can be useful in case of a CD refractory to the GFD.

Screening of asymptomatic first-degree family members of CD patients is recommended. HLA-genotype testing may be carried out as the first-line test; if negative, no further investigation is needed, but if positive, periodic screening with celiac-specific serology should be performed.

#### Management of celiac disease

It is very important to correctly diagnose CD given its excellent response to a GFD (11).

Celiac patients should be educated to avoid food products that contains gluten derived from wheat, barley or rye. On the other side, soybean or tapioca flours, rice, maize and buckwheat are inherently gluten-free and safe. Contamination needs to be avoided, paying also attention to labels on prepared foods and condiments that might contain additives (e.g., stabilizers, emulsifiers) containing gluten.

Separate speech must be made for oats, that should be introduced into the diet with caution, monitoring possible patients’ adverse reactions. Oat consumption should be limited because very likely to be contaminated with wheat/rye/barley: patients can only consume certified gluten-free oats, with crossed grain symbol (19).

Newly diagnosed patients should be referred to a dietitian for discussion of dietary management, evaluation of potential nutrient deficiency, and provision of information on maintaining a strict GFD containing healthy alternatives to gluten (29, 30).

There is evidence that compliance with a GFD is better in those with more knowledge about the diet and CD. Also, the support of health providers and families has a strong positive impact (31).

In recent years, it has been observed that remote monitoring by nutritionists and physicians and point-of-care testing for gluten detection may be helpful in managing patients with CD and checking adherence to a GFD (32).

### Wheat allergy

#### Wheat allergy definition

Wheat allergy (WA) is the clinical expression of an immunologically mediated process that can be immunoglobulin E (IgE) or non-IgE mediated (33). IgE-mediated presentations include: (a) food allergy, (b) wheat-dependent exercise-induced anaphylaxis (WDEIA), (c) baker’s asthma, and (d) contact dermatitis. The non-IgE-mediated, among which CD has been already analyzed, forms include also (a) eosinophil esophagitis, (b) eosinophil gastritis, and (c) dermatitis herpetiformis, where the eosinophilic pathologies are mediated by activation of innate immunity through IL-C2 activity, in contrast to CD and dermatitis herpetiformis which are caused by an immune-mediated mechanism.

Among the different species of wheat, most allergology studies have focused on bread wheat (*Triticum aestivum*) as the marker of wheat sensitivity. Approximately 90% of the wheat cultivated worldwide is bread wheat, while the remaining 10% is durum wheat (*Triticum durum*) which grows well only in the dry climate of Southern Europe and Northern Africa. Recent studies have shown that the allergenic proteins in *T. durum* and *Triticum dicoccum* (spelt) are similar to those in *T. aestivum* and have similar allergenicity in adults and children (34).

#### Epidemiology of wheat allergy

The true prevalence of WA is unknown as self-reported food hypersensitivity overestimates prevalence, but few studies have followed the gold standard method of diagnosis using a double-blind, placebo-controlled food challenge (DBPCFC). Recent studies by Gupta et al. in children and adults estimated prevalence was around 0.5% in children, and around 0.8% in adults, with 52% of cases developing in adulthood. In the USA, wheat is the most common adult-onset food allergy after shellfish (35). Interestingly, prevalence in children can depend upon the timing of wheat introduction, with higher prevalence if wheat is introduced after the age of 6 months (36). However, a high proportion of children tend to grow out of their WA (37, 38). Adult-onset WA seems to have a favorable prognosis, as recently described (39).

#### Clinical aspects

IgE-mediated reactions to wheat can manifest as different phenotypes. Food allergy to wheat can result from gastrointestinal or cutaneous contact, with different clinical presentations, while baker’s asthma is due to sensitization through inhalation.

There are four types of wheat allergy:

(a) Classic wheat food allergy. WA manifests with a variety of symptoms that include urticaria/angioedema, asthma, allergic rhinitis, abdominal pain, vomiting, and anaphylaxis, and is diagnosed following the criteria established by the European Academy of Allergy and Clinical Immunology (EAACI) food allergy and anaphylaxis guidelines (40, 41). Rarely, WA can be a risk factor for severe anaphylaxis and death (42, 43). In children, it can worsen pre-existing atopic eczema.
(b) Wheat-dependent exercise-induced anaphylaxis. WDEIA is a severe type of food allergy (44) that presents during or after intense exercise following wheat ingestion in wheat allergic subjects. Wheat is the most common food causing food-dependent exercise-induced anaphylaxis (FDEIA). Other factors can enhance anaphylaxis such as consumption of non-steroidal anti-inflammatory drugs (NSAIDs) or alcohol. Omega 5-gliadins (also called Tri a 19) are the major wheat allergen responsible for WDEIA. In a limited number of cases, high-molecular-weight glutenin subunits (HMW-GS) also plays a role in WDEIA (45), as well as the wheat lipid transfer proteins (LTP) Tri a 14, especially in Southern Europe. This is a particularly severe form and is frequently associated with anaphylaxis to maize or rice (46).
(c) Baker’s asthma. Baker’s asthma and rhinitis are respiratory occupational diseases caused by sensitization to wheat flours, and are more frequent in atopic subjects (47). Baker’s asthma is one of the most common forms of occupational asthma globally. Regulatory frameworks and workplace control measures should reduce flour dust exposures and related risks. Interestingly, affected subjects can usually safely eat wheat.
(d) Contact dermatitis. Some cases of WDEIA reported between 2009 and 2012 in Japan were caused by mucocutaneous sensitization to hydrolyzed wheat protein added to soap (Glupearl 19) (48). A nationwide investigation revealed that over 2,000 individuals were affected. Interestingly, after the withdrawal of the soap, more than 50% of patients were in remission at 60 months (49).
(e,f) Eosinophilic esophagitis and gastritis. Wheat plays an important role in non-IgE-mediated food allergy, especially in eosinophilic esophagitis and gastritis. These are considered T2 antigen-driven diseases characterized histologically by eosinophilic inflammation and symptoms related to esophageal dysfunction, such as dysphagia, food impaction, and vomiting due to eosinophilic infiltration. Even though atopy is a predisposing condition, IgE antibodies do not seem to play a role in the etiopathogenesis, which is actually driven by innate immunity stimulation through alteration of the mucosal barrier inducing the activation of IL-C2 cells that produce IL-13 and IL-5 responsible for eosinophil recruitment (50).

#### Pathogenesis

The pathogenesis of IgE-mediated WA likely shares the same mechanisms as other food allergies, beginning with a sensitization phase during which wheat allergens are processed by dendritic cells (DC). By interacting with the intestinal epithelial barrier, in the presence of a virus or other irritant stimulus, they cause the release of alarmins, such as IL-33, IL-25, and especially thymic stromal lymphopoietin (TSLP), which induces the expression of OX40 on dendritic cells type 2 (cDC2 103+). These induce the switch from Th0 to Th2 that stimulate B-lymphocytes to produce wheat allergen-specific IgE. The same cDC2 103+ in the absence of damaging stimuli, induce the response of T regs which is the basis of oral tolerance (51). The symptomatic phase is due to the interaction of IgE with mast cells that induces the release of preformed histamine and the production of inflammatory mediators such as prostaglandins, leukotrienes (52), PAF, and cytokines responsible for symptoms.

A recent study focused on the specific mechanism of sensitization to wheat. In a mouse model, skin was sensitized to wheat by repeated skin application of salt-soluble protein extract from durum wheat (53). After 9 weeks, the researchers elicited robust increases in wheat-specific IgE (sIgE) levels and anaphylaxis following oral administration of wheat. This research clearly demonstrates the power of wheat as a food allergen (54). It also supports the dual-allergen exposure hypothesis for the development of food allergies in a mouse model. The authors also demonstrated selective elevation of IL-6 upon oral allergen challenge, suggesting it has a key role in eliciting anaphylaxis *via* the oral route. A similar human model may be represented by the hydrolyzed wheat protein anaphylaxis associated with Glupearl 19 soap as a result of its repeated use, demonstrating sensitization through the skin (55).

#### Wheat allergens

Wheat proteins can be classified into two fractions based on solubility in salt. The salt soluble fraction includes albumins and globulin and represents 15–20% of total proteins. The salt insoluble fraction includes gliadin and gluten and accounts for approximately 80% of wheat protein content (56). Wheat allergens are present in both fractions. A total of 28 wheat allergens have been recognized by the International Union of Immunological Societies (IUIS), several of which are relevant to food allergy and are listed in Table 1.

**TABLE 1:** *Triticum aestivum* (bread wheat) allergens.

Allergen	Biochemical name	MW (SDS-PAGE) (kDa)	Route of allergen exposure
Tri a 12	Profilin	14	Food
Tri a 14	Non-specific lipid transfer protein 1	9	Food
Tri a 19	Omega-5 gliadin	65	Food
Tri a 25	Thioredoxin	25	Food
Tri a 26	High-molecular-weight glutenin	88	Food
Tri a 27	Thiol reductase homolog	27	Airway
Tri a 28	Dimeric alpha-amylase inhibitor 0.19	13	Airway
Tri a 29	Tetrameric alpha-amylase inhibitor CM1/CM2	13	Airway
Tri a 32	1-cys-peroxiredoxin		Airway
Tri a Bd36 kd	Peroxidase	36	Food
Tri a 37	Alpha purothionin	12	Food
Tri a 39	Serine protease inhibitor-like protein		Airway

MW, molecular weight; SDS-PAGE, sodium dodecyl sulfate-polyacrylamide gel electrophoresis.

Profilins (Tri a 12) are one of the allergens underlying cross-reactivity with grass pollen. Recently peroxidase 1 (35 kDa) and beta-glucosidase (60 kDa) were identified as specific IgE-binding wheat proteins cross-reacting with grass pollen allergens (57, 58).

Non-specific lipid transfer proteins such as Tri a 14 from bread wheat and Tri tu 14 from durum wheat are allergens involved in IgE-mediated food allergy, WDEIA, and baker’s asthma, especially in Southern Europe. These forms are also associated to non-IgE–mediated food allergy as demonstrated in a subject with baker’s asthma and CD (59, 60).

Omega-5 gliadin (Tri a 19) have been identified as major allergens in WDEIA. Tri a 25, peroxidase, and serine proteinase inhibitor all have a role in baker’s asthma. Alpha-amylase/trypsin inhibitors are the major allergens in both baker’s asthma and food allergy (56, 61). They are heat resistant and lack significant cross-reactivity to grass pollen allergens. Tri a 37 is a plant defense protein. It is highly stable, resistant to heat and digestion, and a potent allergen (62).

High-molecular-weight glutenins (Tri a 26) are major allergen in WDEIA but have been described in only a few cases (63, 64).

#### Diagnosis of wheat allergy

The diagnosis of WA relies on the demonstration of IgE sensitization and clinical reactivity. Allergic sensitization must be confirmed by *in vivo* (skin prick tests) and/or *in vitro* (specific serum IgE) tests. If severe anaphylaxis has not been documented, it is important to verify symptoms with DBPCFC following the schedules and doses already published (65, 66). The diagnosis of WDEIA is challenging as the usual approach of performing exercise after a wheat-based meal (44) can lead to false-negative results. Brockow et al. analyzed patients with a clinical history of WDEIA and positive omega 5-gliadin-specific IgE levels. In all patients, including some with previous negative wheat challenge results, WDEIA was confirmed by challenge with up to 80 g of gluten alone or with gluten plus cofactors. The authors concluded that higher doses of gluten can elicit a reaction even in the absence of enhancing factors (65). This study seems to demonstrate that IgE positivity to Tri a 19 can be diagnostic for WDEIA, suggesting the importance of component-resolved diagnosis (CRD) in the diagnosis of WA. However, sIgE (and also the skin prick test) cannot distinguish between true WA and sensitization when sIgE to grass pollen is also present, due to the high rate of cross-reactivity between the two allergens (67). In addition to omega-5 gliadin (Tri a 19), which is strictly associated with WDEIA, positivity to Tri a 14, the wheat non-specific lipid transfer protein, is also associated with WDEIA, especially in patients with multiple LTP-containing vegetable food allergies. Given the high rate of cross-reactivity in these cases, a wheat challenge should always be performed (68). Other relevant allergens useful for diagnosis are alpha-amylase/trypsin inhibitors, which are associated in particular with baker’s asthma.

Recent data suggest also a diagnostic role for the basophil activation test (69).

#### Management of wheat allergy

The management of WA varies according to the clinical manifestations and sensitization profile to various allergens. Depending on symptom severity, the diet should be based on either total wheat elimination or the maximum tolerated dose. Although a few patients with food allergy have severe reactions to very small amounts of allergen, most patients can tolerate low doses of the culprit food and may never experience a severe reaction (70). In these cases, consumption of low doses could help them reach tolerance more quickly. An epinephrine autoinjector should be provided to all patients with a history of systemic reactions (44).

Regarding WDEIA, the management of patients with omega-5 gliadin allergy depends on the severity of the reaction (71). Christensen et al. showed that regular intake of wheat not related to exercise can be recommended. The clinical threshold in WDEIA seems to be lowered in patients on a wheat-free diet, while the opposite is seen in patients with regular wheat intake long before exercise (63).

Interestingly, oral immunotherapy has been proposed as a new treatment for WA in children, showing promising results (71, 72).

### Non-celiac (gluten)-wheat sensitivity

The term NCGS was first used in a case report describing the resolution of persistent gastrointestinal symptoms with the adoption of a GFD in a patient in whom CD had been excluded (73).

Non-celiac (gluten) wheat sensitivity is characterized by gastrointestinal and/or extra-intestinal symptoms reported following gluten consumption in subjects not affected by CD or WA (74). Prevalence is difficult to estimate but, in the absence of identified risk factors, NCWS seems to be associated with female gender and young/middle age (75).

The clinical presentation of NCGS/NCWS includes gastrointestinal symptoms (abdominal pain, bloating, and altered bowel habit) and extra-intestinal symptoms, such as fatigue, headache, bone or joint pain, mood disorders, and skin manifestations (e.g., eczema or rash). Reported symptoms usually follow the consumption of gluten and improve after gluten withdrawal (75).

Non-celiac (gluten) wheat sensitivity is a diagnosis of exclusion; the gold standard for diagnosis is a double-step approach as defined by the Salerno Experts Criteria (76). After exclusion of CD and WA, patients start a 6-week gluten-containing diet followed by a GFD for at least 6 weeks. A decrease of at least 30% in the baseline score is considered a positive response. Step 2 includes a 1-week challenge (GFD and gluten or placebo) followed by a 1-week washout period following a strict GFD, and then crossover to the second 1-week challenge. A variation in symptoms of at least 30% between gluten and placebo challenge discriminates a positive from a negative result (76).

### Management of non-celiac (gluten) wheat sensitivity

For diagnosing NCGS/NCWS is essential that testing for CD and WA must be negative, symptoms must improve with a GFD, and diagnosis must be confirmed by the gluten challenge.

However, those who present with self-reported gluten-related symptoms and may have both NCGS/NCWS and secondly those who present with IBS-type symptoms and could have gluten or wheat sensitive IBS. Those patients report that food plays an important role in their IBS-type symptoms with estimates of up to 80% of patients having postprandial symptomology, and up to 40% reporting specific “food intolerances.” The fundamental difference between NCGS/NCWS and IBS is that patients with NCGS/NCWS self-report symptoms when consuming gluten and have identified or perceive gluten as the culprit. Conversely IBS patients do not report gluten as a specific stimulus for their symptoms. However, previously previous studies have demonstrated that wheat is a commonly reported “food intolerance” when IBS patients are specifically questioned (12).

There is insufficient evidence to determine whether NCGS/NCWS is a chronic relapsing/remitting disease or self-limiting disease and how strict the diet needs to be. A trial of gluten reintroduction can be attempted in individuals who remain asymptomatic for a period of time; however, symptoms with reintroduction are likely to be extremely variable among individuals (28). Therefore, once the diagnosis of NCGS/NCWS is reached, the management and follow-up of patients are not standardized, because of the unclear pathogenesis and variable clinical presentation (30, 76). The authors suggest that a logical approach could be to undertake a GFD for a limited period, followed by the gradual reintroduction of gluten. After some period on a GFT, the reintroduction of gluten could start with cereals of low gluten content (e.g., oats). Chronically, periods of GFD could be suggested during symptomatic relapses as a sort of “on-demand” therapy, also considering other possible treatments when there is the suspicion of a wheat sensitive IBS.

## Discussion

The diagnosis of CD and WA is objective, albeit difficult. nonetheless the Patient with symptoms can find a pathological explanation for them, and a specialist to whom they could refer for their disease (the gastroenterologist with the nutritionist or the allergologist), and the most effective therapy, even in the case of refractory CD or severe WA. However, there are no clear serological or histopathological criteria to confirm a diagnosis of NCGS/NCWS since antibodies (tTG-IgA and EMA-IgA) indicating CD are negative in NCGS/NCWS and histology does not show signs of atrophy.

In addition, patients with NCGS/NCWS may believe they do not have disease or conversely, that clinicians think they are imaging it. NCGS/NCWS has often been compared to functional diseases and described as an irritable bowel syndrome (IBS)-like entity (12, 77). Indeed, NCGS/NCWS has been reported as wheat-sensitive irritable bowel syndrome when fulfilling the criteria for IBS according to the Rome Foundation (12).

Furthermore, symptoms sometimes improve in patients with either NCGS/NCWS or IBS after a reduction in FODMAP intake (fermentable, oligo-, di-, monosaccharides, and polyols) (78). FODMAPs enter the colon where they are fermented, causing the production of gas and since they are osmotically active, they can lead to increased water content in the intestinal lumen. This process is thought to be amplified in the presence of intestinal dysbiosis and may cause symptoms such as abdominal pain, diarrhea, flatulence, and bloating (79). A GFD is often recommended for patients with self-reported gluten/wheat-dependent symptoms, but a low-FODMAP diet should be the first option for patients with symptoms similar to those of functional diseases. In these patients, evidence may justify the prescription of a low-FODMAP diet because of its demonstrated beneficial effects In particular, three meta-analyses have demonstrated that a low FODMAP diet improves global symptoms in IBS patients compared with other dietary interventions (80–82). A recent randomized controlled study in patients with IBS with diarrhea (IBS-D) evaluated the efficacy and acceptability of a short-term strict low FODMAP diet and of a long-term modified FODMAP diet compared with traditional dietary advice, and showed that both the strict and modified low FODMAP diets are acceptable and lead to significant improvements in symptoms and quality of life (83). United European Gastroenterology (UEG) guidelines on functional bowel disorders with diarrhea recommend the short-term use of a low FODMAP diet in patients with IBS-D when other measures have failed (84).

The complexity of the diet requires the involvement of experienced nutritionists (83, 85–87).

Nutritionists and physicians should clearly explain to patients referring all his symptoms to gluten ingestion that it is likely that in addition to gluten, other non-gluten wheat components, e.g., fructans, yeasts (which are scientifically known as FODMAPs) may also be the responsible for triggering symptoms.

It is likely that in some patients the ingestion of gluten-containing foods may have a nocebo effect, while in others the avoidance of gluten-containing foods could have a placebo effect (88). When wheat-sensitive IBS is suspected, also non-diet specific treatments should be considered, such as neuromodulators, probiotics, and physical activity (84, 89, 90).

In conclusion, a personalized approach, regular follow-up, and the help of a skilled nutritionist are mandatory for patients reporting symptoms after wheat ingestion.

## Author contributions

AC, GA, GL, VS, and EP wrote the manuscript. LD reviewed the manuscript. LE, EP, LR, and MV supervised the study and critically reviewed the manuscript. All authors contributed to the article and approved the submitted version.
